# Liposome retention in size exclusion chromatography

**DOI:** 10.1186/1472-6750-5-11

**Published:** 2005-05-10

**Authors:** Tristan Ruysschaert, Audrey Marque, Jean-Luc Duteyrat, Sylviane Lesieur, Mathias Winterhalter, Didier Fournier

**Affiliations:** 1 Groupe de biotechnologie des protéines, Institut de Pharmacologie et de Biologie Structurale, F-31077, Toulouse, France; 2 Electron Microscopy Department, Rangueil Hospital Medical School, University of Toulouse, 31062 Toulouse, France; 3 Groupe Physico-chimie des systèmes polyphasés, Université Paris-sud, F-92296, Châtenay-Malabry, France; 4 International University Bremen, D-28726 Bremen, Germany

## Abstract

**Background:**

Size exclusion chromatography is the method of choice for separating free from liposome-encapsulated molecules. However, if the column is not presaturated with lipids this type of chromatography causes a significant loss of lipid material. To date, the mechanism of lipid retention is poorly understood. It has been speculated that lipid binds to the column material or the entire liposome is entrapped inside the void.

**Results:**

Here we show that intact liposomes and their contents are retained in the exclusion gel. Retention depends on the pore size, the smaller the pores, the higher the retention. Retained liposomes are not tightly fixed to the beads and are slowly released from the gels upon direct or inverted eluent flow, long washing steps or column repacking. Further addition of free liposomes leads to the elution of part of the gel-trapped liposomes, showing that the retention is transitory. Trapping reversibility should be related to a mechanism of partitioning of the liposomes between the stationary phase, water-swelled polymeric gel, and the mobile aqueous phase.

**Conclusion:**

Retention of liposomes by size exclusion gels is a dynamic and reversible process, which should be accounted for to control lipid loss and sample contamination during chromatography.

## Background

Liposomes are self-assembled phospholipids enclosing a droplet of the aqueous medium in which they are formed [1]. Liposomes have numerous applications namely as *in vivo *drug delivery vehicles [2]. Drugs interact with liposomes in several different ways depending on their solubility and polarity characteristics. They can be inserted in the lipid chain bilayer region, intercalated in the polar head group region, adsorbed on the membrane surface, anchored by a hydrophobic tail or entrapped in the inner aqueous compartment. A prerequisite for the use of drug-loaded liposomes is to be able to separate encapsulated from free drugs. Recently, we encapsulated enzymes in liposomes to enhance the enzyme stability with respect to dilution and proteases [3,4]. Size exclusion chromatography (SEC) is an old and widely used tool to separate small solutes from liposomes or to narrow the size distribution [5,6]. For example, Sepharose 2B, 4B Sephacryl S-1000 and high performance exclusion gels of the TSK-PW series are suitable for separating small unilamellar vesicles from larger ones [6-10]. In 1977, Sorensen et al. [11] showed loss of lipid material during SEC of liposomes. In 1980, Van Renswoude et al. [12] observed by microscopy the retention of fluorescent liposomes between and on the polymer beads. The loss of lipids could reach 20 to 40% of the deposited material [8,13]. These findings suggested a step of gel pre-saturation with lipids to avoid loss during SEC [14]. To achieve high quality separation, the column pre-treatment is preferentially carried out with sonicated liposomes as their small sizes ensure efficient penetration of the lipids within the gel pores [15].

Here we show that when liposomes containing a hydrophilic protein pass through a SEC column, lipids and protein are simultaneously retained suggesting that the liposomes retained remain whole. Retention is, however, transitory and reversible: there is an equilibrium between retained liposomes and eluting ones. A pre-saturation step should thus be performed with liposomes of the same composition as that of the liposomes to be chromatographied in accordance with previous recommendations [10].

## Results

### SEC elution pattern of liposomes

**Figure 1:**
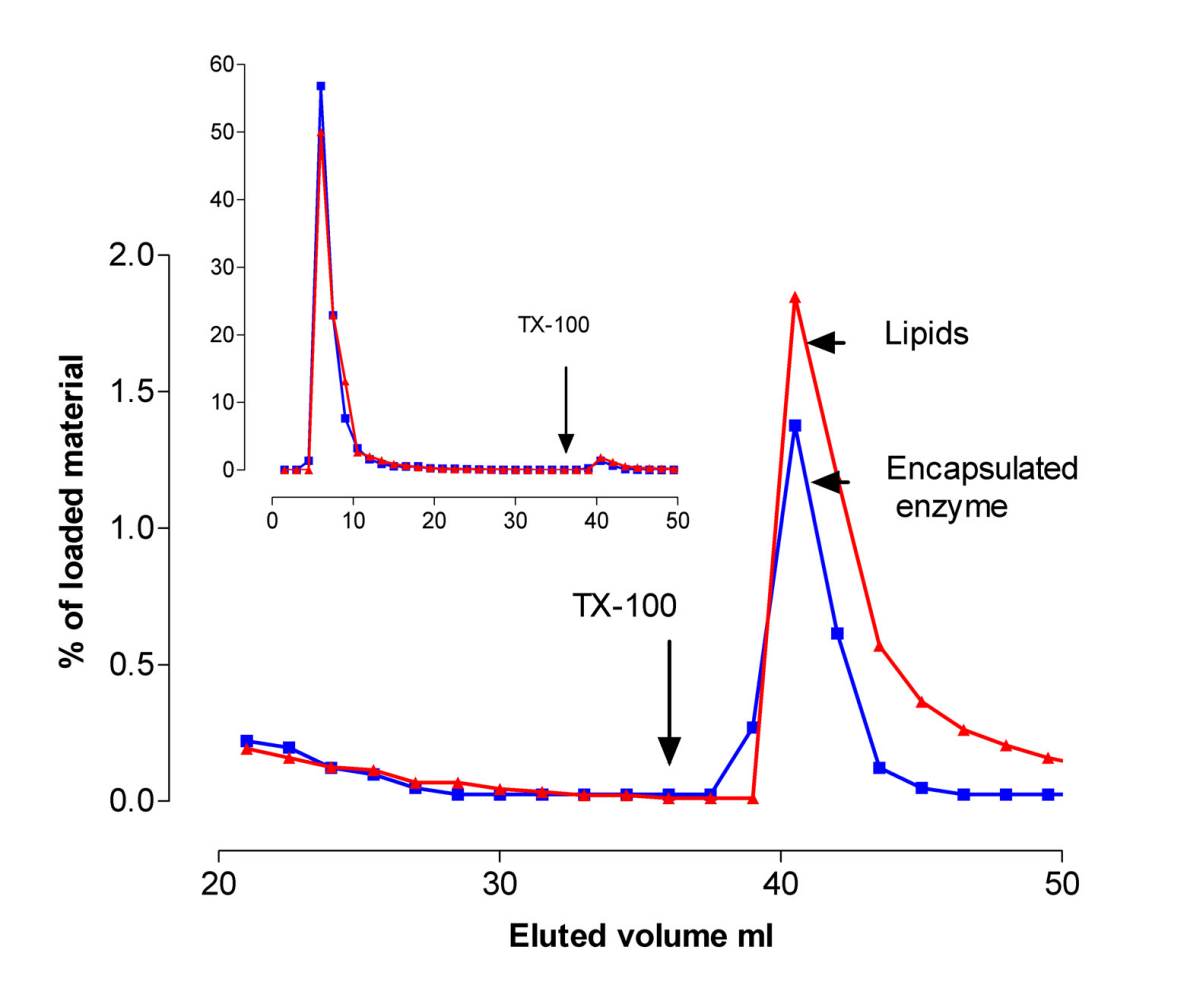
**Elution pattern of lipids and encapsulated enzymes following liposome chromatography on G25 column. **Liposomes labeled with rhod-PE and containing AChE were chromatographied on G25M. Fractions of 1.5 mL were collected. Fluorescence (in red) and enzyme activity (in blue) were measured as a percentage of the total fluorescence or enzyme activity collected. Arrows indicate the injection of TX-100 on the top of the columns. The general elution profile is represented in the inset.

In order to quantify the amount of lipid retained on a freshly prepared column (not previously saturated with lipids) we injected 400 μL liposomes incorporating 1 mol % Rhod-PE as fluorescent label and loaded with 5 to 10 acetylcholinesterase molecules as internal material. The sample was passed through an 8 mL G25 column. The majority of lipid and enzyme was excluded from the pores of the gel beads and eluted together at the void volume of the column (from 6 to10 mL, Fig. 1). To determine the amount of lipid and enzyme retained in the gel, TX-100 was added to the elution buffer such that the final eluent concentration was 0.5% (w/v). This caused co-elution of a significant amount of lipid and enzyme. Control measurements performed either with free enzyme, or with a mixture of free enzyme and empty liposomes, did not show any retention of enzyme under similar conditions. A further experiment was performed with calcein-loaded liposomes. Identical results were obtained: TX-100 treatment led to the concomitant elution of lipids and calcein under a broader peak than in the case of enzyme-loaded liposomes, due to the smaller molecular mass of calcein (see below). Retention of lipids and of hydrophilic encapsulated materials were identical, suggesting that intact liposomes were retained during SEC.

### Column saturation

**Figure 2:**
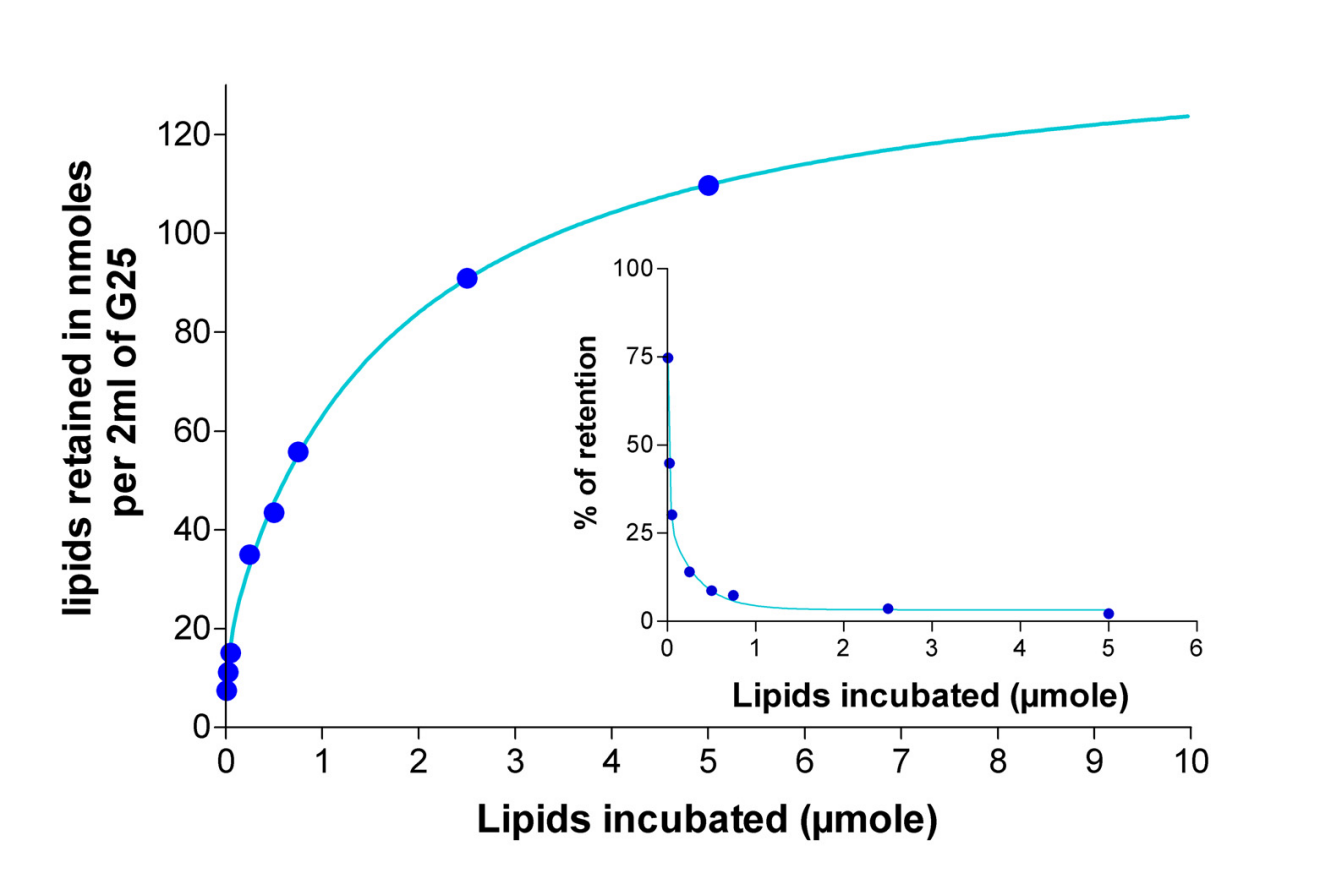
**Liposome retention depends on the amount of lipids loaded. **Liposomes labeled with Rhod-PE were incubated with 2 mL G25M gel two hours with gentle agitation and poured in columns. Non-retained liposomes collected by washing the column with 40 mL buffer and retained liposomes were eluted by adding 0.5% TX-100.

We observed lipid retention when the chromatography gel was incubated with liposomes prior to being poured into the column. For convenience, we used this method to characterize the relationship between the amount of lipid added to the gel and the amount retained. 2 mL of swollen G25 were incubated in 5 mL buffer containing various concentrations of Rhod-PE labeled liposomes to evaluate the parameters underlying liposome retention. Apparently lipid retention onto the gel beads shows a saturation limit (Fig. 2). It appears that saturation is obtained by passing at least 5 μmole of lipids per ml gel.

### Relationship between retention of lipids and encapsulated enzyme

**Figure 3:**
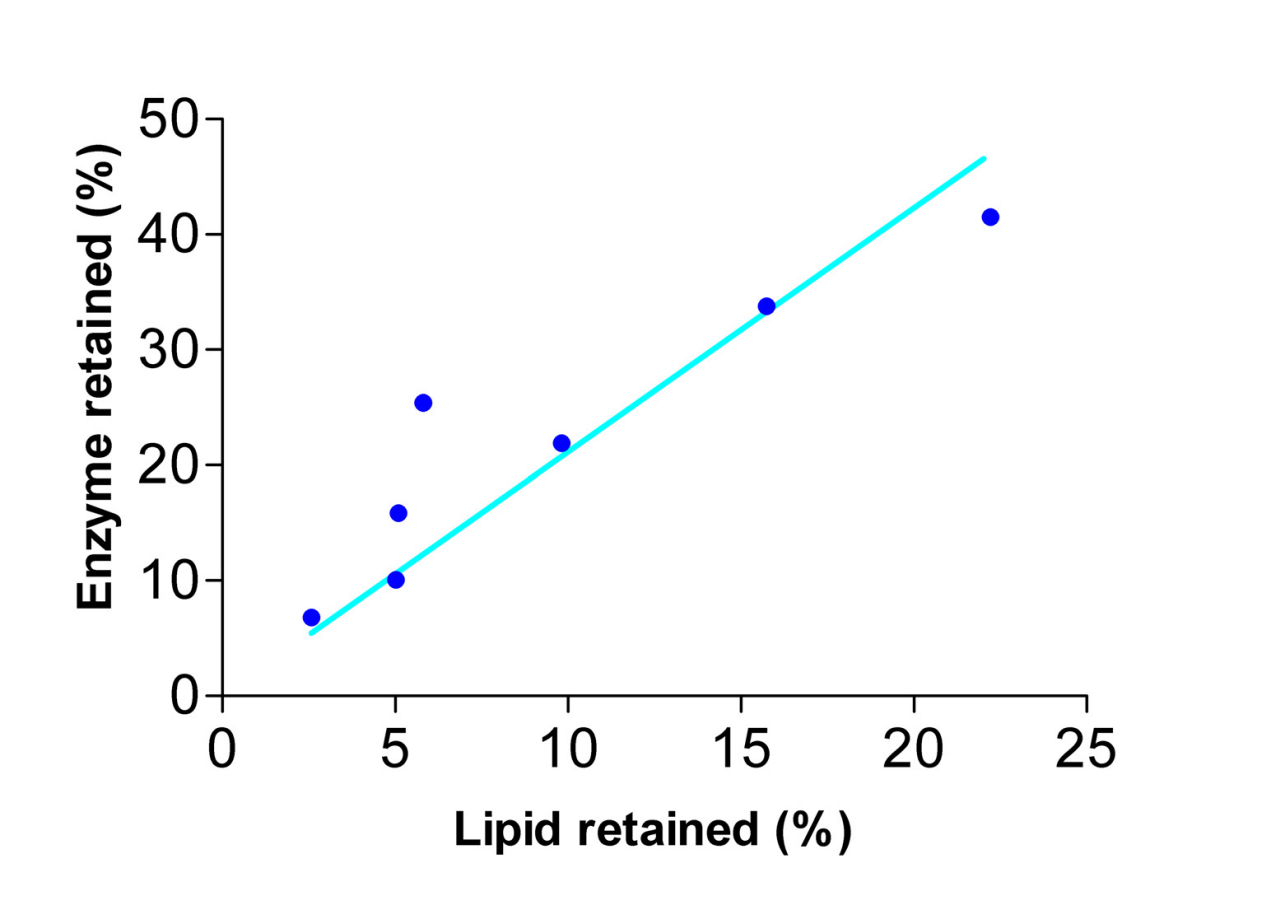
**Relation between lipid and intravesicular marker retention. **Liposomes labeled with rhod-PE and containing AChE were chromatographied on G25M. Fluorescence and activity were collected before and after addition of TX-100.

Liposomes loaded with AChE were incubated with 5 mL of fresh G25 in 9 mL buffer. Different concentrations of liposome were used to obtain different retention efficiencies. After 2 h, the gel was poured into a column, washed and eluted with TX-100. The lipid and enzyme content of the fractions were quantified. We observed a linear correlation between lipid and protein retention (Fig. 3). In a control experiment, when liposomes and free enzyme were loaded together on the column, no enzyme retention was observed. This suggests that retention of membrane and intravesicular content are linked, consistent with the hypothesis of non-damaged liposome retention. Furthermore, we observed greater retention of AChE than of lipids suggesting liposome reorganization during the washing step.

### Retention depends on gel exclusion limits

**Figure 4:**
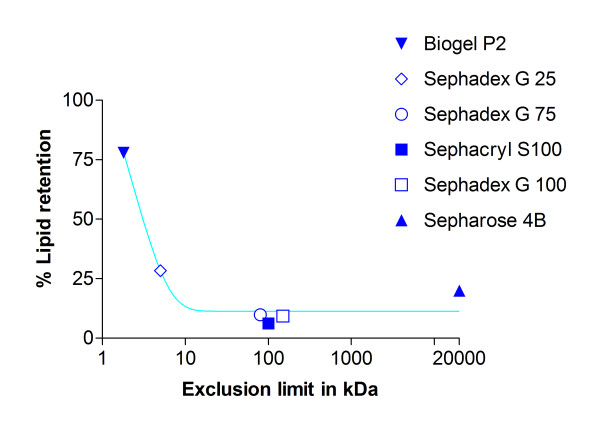
**Liposome retention depends on bead pore size. **Liposomes labeled with Rhod-PE were incubated with different gels and poured into columns. Fluorescence was collected before and after TX-100 addition to estimate the proportion of liposomes retained.

Labeled liposomes were incubated with different gels and retention was estimated by elution with TX-100. Retention appeared to depend on the pore size of the beads responsible for the size exclusion (Fig. 4). The smaller the pore sizes, the greater the amount of liposomes retained. The exclusion limit of the gels in our study was significantly smaller than the liposomes size. Exception was noted for Sepharose 4B, this gel reached the range of 60 nm diameters exclusion limit, which is enough to allow small liposomes to penetrate the pores of the SEC gel and to obey an effective permeation process. This chromatography may explain the apparent higher retention than expected. On the other hand, retention was independent of the size of the gel beads since G25 beads of different sizes, fine (20–80 μm diameter), medium (50–150 μm) and coarse (100–300 μm) exhibited the same retention efficiency (data not shown).

### Elution by extensive washing step, flow inversion or column repacking

**Figure 5:**
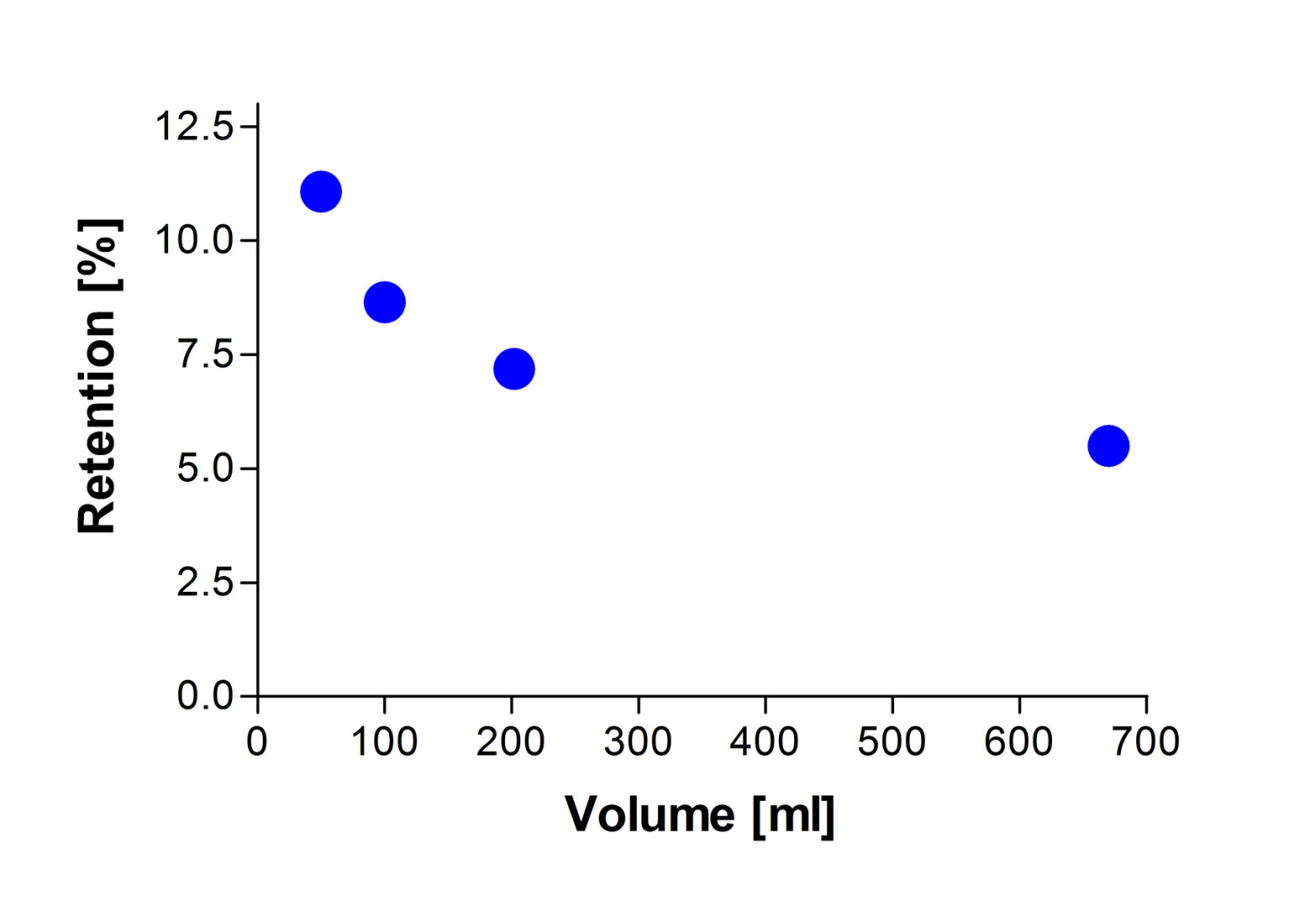
**Elution of retained liposomes by the buffer flow. **Liposomes labeled with rhod-PE were chromatographied on 8 mL G25M. TX-100 was added after different extensive washing volumes to estimate the slow elution due to shearing forces of the buffer flow.

In order to distinguish binding from kinetic trapping, we modified the washing volume. 400 μL of liposome solution were loaded onto 8 mL G25 gels and washed with different amounts of buffer. The remaining retained liposomes were quantified with TX-100 elution. Fig. 5 shows that retention decreased as the washing step was increased.

**Figure 6:**
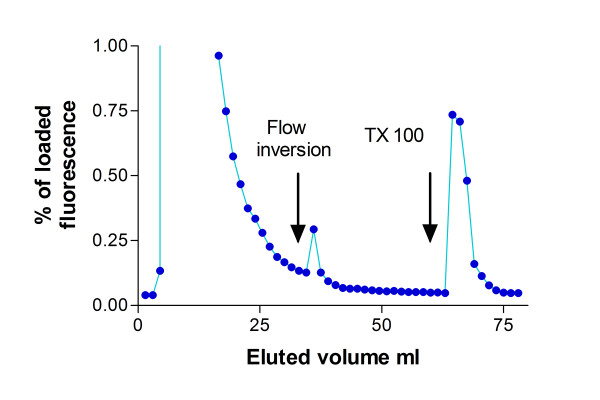
**Flow inversion eluted a part of the retained liposomes. **Liposomes were chromatographied on G25M. Fractions of 1.5 mL were collected. First arrows indicate the inversion of buffer flow and the second one the injection of 0.5% TX-100 on the top of the column.

To test if liposomes were trapped between the beads due to column packing, elution was applied backwards. First 400 μL of Rhod-PE labeled liposomes were loaded on 8 mL G25 and washed. After exclusion of non-retained liposomes, the buffer flow was inverted. The elution profile (Fig. 6) showed that some lipids were eluted by the flow inversion. However, only a small proportion of the retained liposomes were eluted as evidence by the amount of lipids eluted by adding TX-100. Identically, depacking a gel and pouring it into a column once more resulted to partial elution of the retained liposomes.

### Direct observation by scanning electron microscopy (SEM)

**Figure 7:**
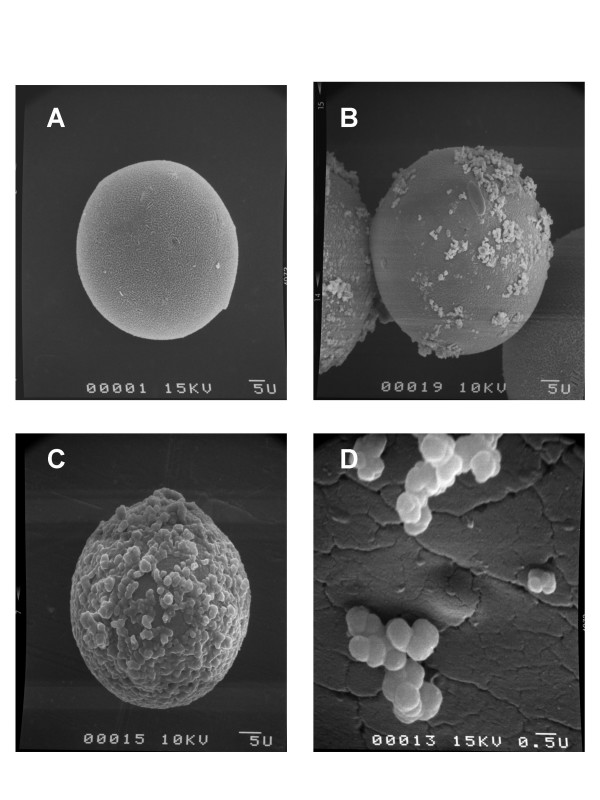
**SEM observation of retained liposomes on G-25 fine beads**. A : Sephadex beads non incubated with liposomes, fixed with glutaraldehyde, washed, stained and prepared for SEM. B, C, D: Liposomes, containing PC and PE (6/4), retained by a 2 mL sephadex G-25 fine columns were fixed by glutaraldehyde, washed, stained and prepared for SEM observations.

SEM photographs of sephadex beads were performed to observe how lipids are retained in SEC columns and to investigate how lipids bind to the beads, as individual molecules, as membranes or as liposomes at the surface or inside the beads. Liposomes containing 40% of PE and extruded at 200 nm were passed onto a Sephadex G-25 fine column. After intensive washing, samples diluted in Sorensen buffer were fixed with 2% glutaraldehyde. Without liposomes, the beads appeared homogeneous (Fig 7 A). In contrast, beads originating from the column were covered by lipid aggregates resembling liposomes. Thus, it seems that entire liposomes are retained on the beads. Some liposome aggregation appeared on the beads which may result from the glutaraldehyde cross-linking. Liposomes appeared to be larger than expected with a 500 nm diameter compared to the 250 nm estimated by dynamic light scattering before loading. This difference suggests that liposomes fused either during the chromatography process or during fixation. It also appeared that some beads retained few liposomes (Fig 7 B) while others were completely covered (Fig 7 C). This heterogeneity might result from heterogeneity of column saturation from the top to the bottom.

### Elution of retained lipids by liposomes in the mobile phase

**Figure 8:**
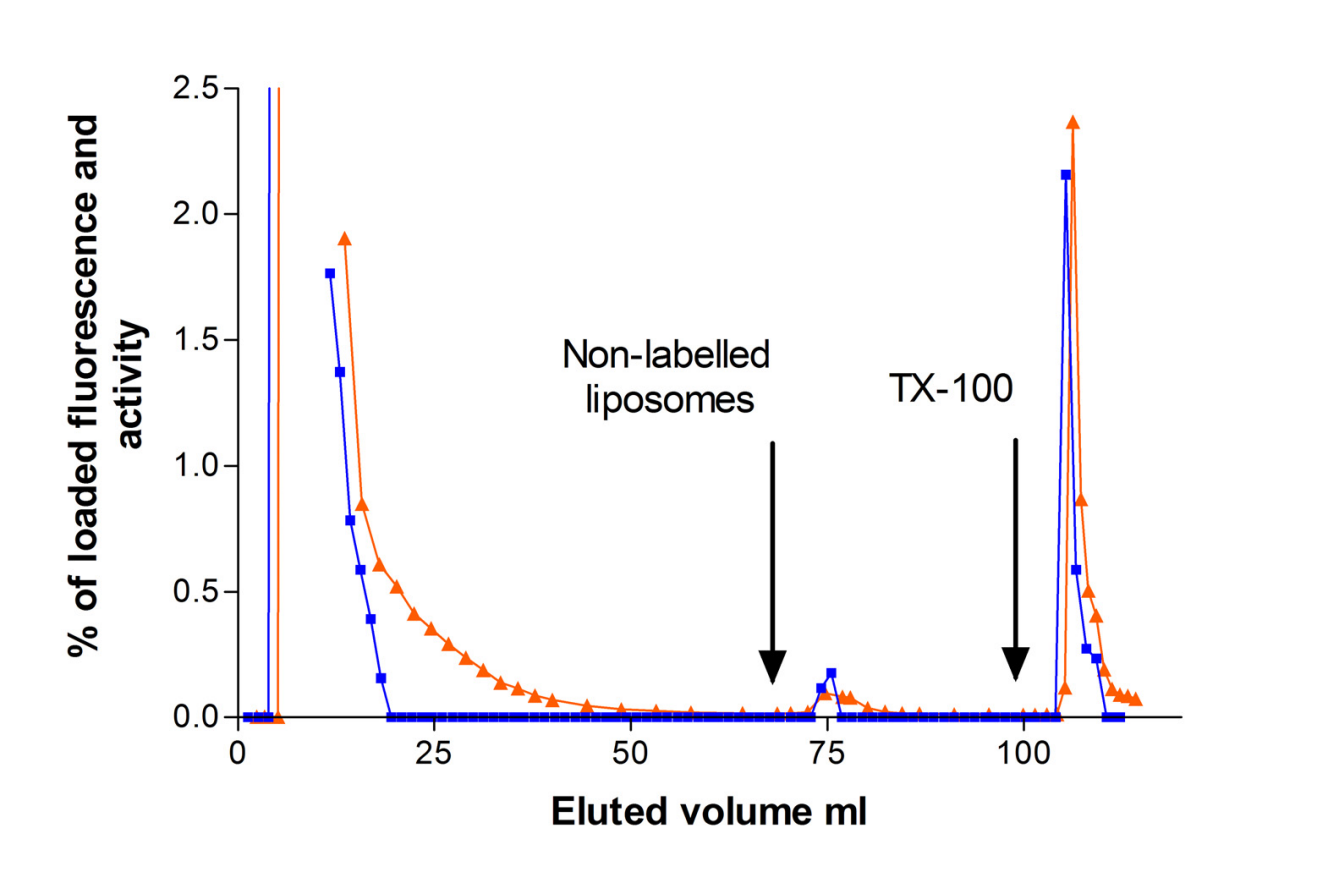
**Elution of retained labeled liposomes with Rhod-PE and AChE by passing non-labeled liposomes**. Liposomes, labeled with rhod-PE (fluorescence represented with red triangles, 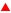) and containing AChE (activity represented with blue squares, 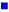) were chromatographied on G25M. Fractions of 1.5 mL were collected. The first arrow indicates the injection of unlabeled liposomes on the column and the second arrow indicates the injection of 0.5% TX-100 on the top of the columns.

**Figure 9:**
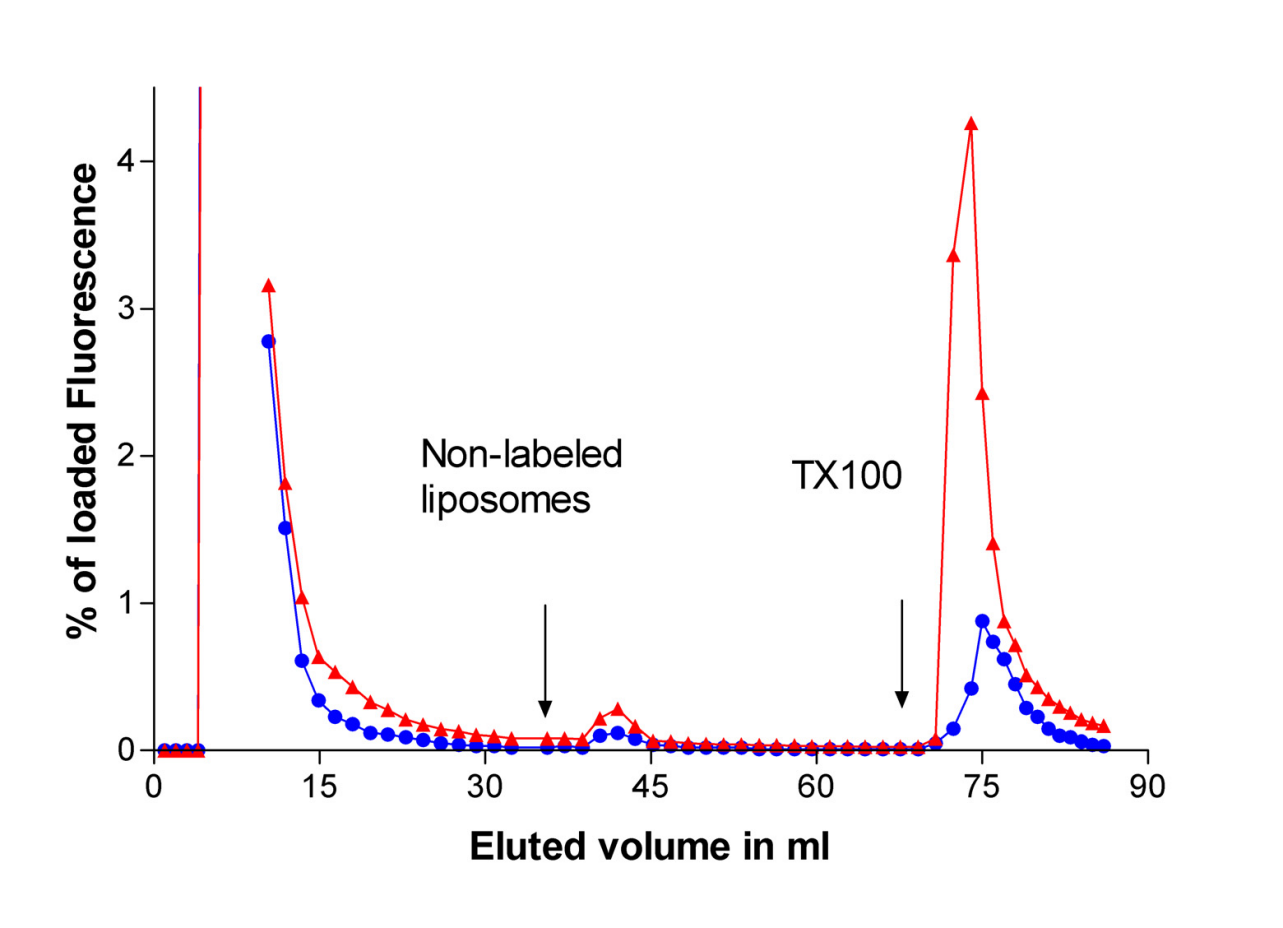
**Elution of retained labeled liposomes with Rhod-PE and calcein by passing non-labeled liposomes**. Liposomes, labeled with rhod-PE (fluorescence represented with red triangles, 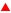) and containing calcein, 500 μM (fluorescence represented with blue circles, 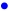) were chromatographied on G25M. Fractions of 1.5 mL were collected. The first arrow indicates the injection of unlabeled liposomes on the column and the second arrow indicates the injection of 0.5% TX-100 on the top of the columns.

We tested if liposomes could be dissociated from the column by other liposomes. Liposomes labeled with fluorescent lipids and containing AChE, were loaded on a G25 column. Following exclusion of non-retained liposomes, unlabeled liposomes were loaded on the column. It appeared that some retained lipids and enzymes were co-eluted by the unlabeled liposomes (Fig. 8). Enzymes eluted by liposome were encapsulated because their activity was detectable only by using TX-100 in the solution. TX-100 disrupted liposome bilayers and allows the enzyme substrate to reach AChE which is unable to cross the lipid membrane [16]. The same experiment was performed with an intravesicular tracer of smaller size: calcein. This fluorescent probe was loaded in rhod-PE labeled liposomes. The elution profile (Fig. 9) shows one peak for excluded liposomes. Passing unlabeled liposomes resulted in an elution of both retained labeled lipids and calcein.

In order to test lipid exchanges between liposomes in the column, the previous experiment was repeated replacing the unlabeled passing liposomes by NBD-PE labeled liposome on Rhod-PE ones retained in the gel. As previously, passage of NBD-PE labeled liposomes, led to the elution of Rhod-PE liposomes. In addition, we observed elution of NBD-PE liposomes by adding TX-100. Thus, it appears that binding and release of liposomes on Sephadex columns is a dynamic process.

## Discussion

### What is retained, lipids or liposomes?

Lipid retention has been observed since the beginning of the 80's [12] and usually pre-saturation steps are used to avoid this phenomenon. However, a question remained: are liposomes or lipids retained? To address this, we compared the retention of lipids and the retention of encapsulated materials, enzyme or calcein. It appeared that intravesicular material is also retained suggesting that lipids are retained as intact liposomes without leakage (Fig. 1, 3 and 9). Furthermore SEM observations of the beads clearly showed the presence of lipid aggregates resembling liposomes, attached to the beads (Fig. 7). The next question should be: why are liposomes retained in SEC gels, whereas their size and shape would predict their exclusion? A first hypothesis is a direct interaction of lipids with the beads at the liquid/solid interface of water and the poly-dextran beads, this interaction may promote lipid reorganization as in liquid/air monolayer experiments. As full liposomes are retained in columns, this hypothesis seems less probable because such an interaction would destroy the liposome integrity and its contents would be released. A second hypothesis arises from liposomes' flexibility: they may be deformed and thereby pass through the polymer net and remain stuck [17]. Several experiments favor this hypothesis. i) Retention depends on the bead pore size (Fig. 4) ii) Partial elution can be performed by inversion of the buffer flow (Fig. 6), depacking and repacking the gel in the column or extensive washing (Fig 5).

### Is it possible to saturate a column to avoid liposome retention?

Figure 2 shows a saturation plateau at 60 nmoles lipids per mL of G25 suggesting that once the column has been saturated, SEC can be performed without liposome retention. However, if all the pores are filled with liposomes, like in the picture in fig 7C, the efficiency of chromatography may be affected. Furthermore, saturation depends on the washing step, the longer it is, the less the column remains saturated. Saturation is not static but seems to be the result of a dynamic process resulting from exchanges between free and entrapped liposomes. In conclusion, saturation of a column should be used with caution bearing in mind the reversibility of the saturation process, which can cause contamination of the samples to be analyzed.

### Interaction of liposome with beads and leakage

Two observations suggest that retention of liposomes inside the beads is associated with liposome reorganization. First, retention of enzyme appeared to be higher than lipid retention suggesting that entrapped liposomes lose their lipids (Fig. 3) and second, free liposomes passing through the column are able to elute fixed liposomes (Fig. 8 and 9), most probably by fusion and fission. Is there some leakage of encapsulated molecules during SEC? Andersson and Lundahl [18] hypothesized leakage of glucose from the liposome due to interaction between the liposomes and the gel bead surfaces, and disruption of liposomes by shear forces from the liquid flow. However, simple permeation of glucose across the lipid bilayer is significant and does not allow permeation to be distinguished from leakage during the several minutes needed to perform SEC. However, our enzymatic test did not provide evidence of any liposome leakage or internal content release with time. This strongly supports that the transitory retention of lipids does not induce dramatic vesicle leakage or bilayer permeability enhancement and rather agrees with intact liposome trapping.

### Relation with immobilized-liposome chromatography

Immobilized-liposome chromatography is used for studying the partitioning of compounds into phospholipid bilayers. Several methods are used to bind liposomes to the matrix. Specific ligands such as hydrophobic ligands [19] can be coupled to the gel beads. Liposomes can be formed in the presence of beads using the detergent dialysis method. In this method, lipids, detergent and beads are mixed and dialyzed. Elimination of detergent molecules during the dialysis results in the formation of liposomes entrapped in the beads [19]. A third method comes close to liposome chromatography: beads are mixed with liposomes and binding is achieved by several cycles of freezing and thawing [20]. Entrapped liposomes are thought to be suspended in micro cavities of gel beads [20]. Our results suggest that part of the liposomes spontaneously rearrange in gel beads without outer stress. Buffer flow would be sufficient to push the liposomes inside the bead net and to immobilize them.

## Conclusion

How should SEC be performed with liposomes? As retention is inversely related to the bead exclusion limit, the larger-pore gels should be preferred for liposome separation from small molecules as suggested by Grabielle-Madelmont et al [15]. As retention is proportional to the gel volume, the bed volume of the column has to be calculated at minima for optimal separation, which also avoids sample dilution. The pre-saturation step diminishes liposome retention but as there are liposome exchanges, the purity of the sample may be affected if the retained liposomes are different from the free liposomes. Two ways are possible: (1) for analytic purposes, it is thus better to inject the liposomes to be sized several times until gel saturation and then perform size analysis (2) for preparative purposes like separation of loaded liposomes from non-encapsulated material, the problem is trickier and the experimental conditions should be adapted to minimize loss of lipid and contamination.

## Methods

### Materials

The lipids, egg-phosphatidylethanolamine (PE), brain Lα-phophatidylserine (PS), Lα-phosphatidylethanolamine-N-(lissamine-rhodamine-B-sulfonyl) (Rhod-PE), Lα-phosphatidylethanolamine N-(4-nitrobenzo-2-oxa-1,3-diazole) (NBD-PE), were purchased from Avanti Polar Lipids, Birmingham, AL, USA. Free rhodamine or free NBD which may contaminate labelled lipids were eliminated from liposome solution by chromatogaphy or dialysis. Egg-phosphatidylcholine (PC), was from Lipoid, Ludwigshafen, Germany. Calcein (high purity) was from molecular probes, Leiden, the Netherlands. Sephadex G-25 Medium, G-75 Medium and G-100 Medium, Sephacryl S-100 HR and Sepharose 4B were from Amersham Biosciences, Biogel P2 (45–90 μm) was from Bio-Rad.

Unless otherwise stated, all experiments were performed using 145 mM NaCl, 2.5 mM HEPES, pH 7.4 buffer as aqueous phase. Chromatography gels were washed before use by 0.5% Triton X-100 (TX-100) (Merck), water and equilibrated with buffer.

### Liposome preparation

1 μmole egg-PC dissolved in CHCl_3 _was placed in a 10 mL glass tube. Then this was dried under a stream of N_2 _and under vacuum for three hours to form a dry lipid film. 200 μL of buffer were added to the lipid film and vortexed to peel off the lipid. The liposome suspension obtained was frozen in liquid N_2 _and thawed in a 25°C water-bath 10 times. Buffer was then added to obtain a 1 mM lipid dispersion, which was passed 10 times through a 0.2 μm polycarbonate filter (Schleicher & Schuell, Dassel, Germany). Labeled liposomes were prepared according to the same procedure except that the initial lipid film was obtained from a mixed chloroform solution of egg-PC and 1 mol% Rhod-PE or NBD-PE. Liposome size was estimated to be 230 nm (diameter) by dynamic light scattering.

### Fluorescence

To follow lipid elution in SEC, we measured the lipid content in each fraction by two methods: a phosphate assay to measure all lipids and a fluorescent assay to measure labelled lipids. As we found a good correlation between the two methods, we used only the second one allowing studies with lower amount of lipids to analyze retention of liposomes. Rhod-PE (excitation wavelength 550/emission wavelength 590 nm) and NBD-PE (excitation wavelength 470/ emission wavelength 530 nm) were used as membrane fluorescent markers. Calcein was used at 500 μM at an excitation wavelength of 492 nm and emission wavelength of 517 nm. Fluorescence was measured using a lamp LPS220 and a photomultiplier detection system PDS 810 from photon Technology International.

### Enzyme encapsulation

To obtain liposomes loaded with 5–10 enzyme molecules, 1 μmole of dried eggPC was dissolved with 200 μL of enzyme solution containing 4 nanomoles of *Drosophila melanogaster *acetylcholinesterase (AChE) in buffer. The liposome dispersion obtained was frozen in liquid N_2 _and thawed in a 25°C water-bath 25 times to allow the entrance of the protein [3,4]. Non-encapsulated AChE was removed by inverse affinity chromatography, passing the liposome dispersion through a column filled with procainamide gel that retains free AChE. Elution contained at least 90% encapsulated enzyme and less than 10% free enzyme. Liposome containing fractions were pooled and lipid concentration was adjusted to 1 mM. Extrusion was performed 10 times through a 0.2 μm filter. AChE activity was measured with the sensitive method of Ellman [21] with 1 mM acetylthiocholine after dissolution of liposomes with 0.1% (w/v) TX-100.

### Calcein encapsulation

To obtain liposomes loaded with calcein, 1 μmole of dried eggPC was dissolved with 1 mL of buffer containing 500 μM of calcein. The liposome dispersion obtained was frozen in liquid N_2 _and thawed in a 25°C water-bath 25 times to allow the entrance of the calcein and extrusion was performed 10 times through a 0.2 μm filter. Non-encapsulated probe was removed by passing the liposomes through a Sephadex G-100 column. The fluorescence of the fractions was measured in the presence of TX-100 to avoid calcein self-quenching.

### SEC preparation

1 cm diameter columns were used with two different methods. In the first one, the gel was freshly packed and the liposomes were deposited when the eluent level reached the top of the gel. Buffer flow was driven by simple gravity. The sample was allowed to penetrate the gel entirely before adding repetitive small buffer volume to proceed to the elution. In the second one, swollen gel was incubated in a buffer containing liposomes with gentle agitation for two hours. The gel was then packed into the column and non retained liposomes were eluted by successive additions of small amounts of buffer.

### Sample preparation for scanning electron microscopy (SEM)

For SEM analysis, liposomes containing 60% PC and 40% PE were prepared as previously described. PE was chosen to allow cross-linking by glutaraldehyde. After passing and washing the liposomes on G-25 fine beads were fixed with 2% glutaraldehyde in 0.1 M cacodylate buffer (pH 7.4) (Sorensen buffer) containing 6 mM CaCl_2_, for 4 h, at 4°C. After an extensive wash in the same buffer, samples were removed, post-fixed for 1 h at room temperature with 1% osmium tetroxide in 0.1 M cacodylate buffer (pH 7.4), dehydrated in a graded ethanol series, dried by critical point drier with an EMSCOPE CPD 750 and coated with gold-palladium for 3 minutes at 100 Ångströms / minute, and observed with a S-450 scanning electron microscope (Hitachi) at an accelerating voltage of 10 kV.

## List of abbreviations

AChE: Acetylcholinesterase

NBD-PE: Lα-phosphatidylethanolamine N-(4-nitrobenzo-2-oxa-1,3-diazole)

PC: Egg-phosphatidylcholine

PE: Phosphatidylethanolamine

Rhod-PE: Lα-phosphatidylethanolamine-N-(lissamine-rhodamine-B-sulfonyl)

SEC: Size Exclusion Chromatography

SEM : Scanning Electron Microscopy

TX-100: Triton X-100

## Authors' contributions

TR and AM carried out the SEC studies. JLD carried out the preparation and the observation of samples by SEM. SL and MW participated in the design of the study and in the data analysis. DF conceived coordinated the study.. All authors read and approved the final manuscript.
